# New insights into the structural characteristics of irradiated crotamine

**DOI:** 10.1186/s40409-015-0013-z

**Published:** 2015-05-20

**Authors:** Karina Corleto Oliveira, Patrick Jack Spencer, Rui Seabra Ferreira, Nanci Nascimento

**Affiliations:** Nuclear and Energy Research Institute (IPEN/CNEN-SP), Avenida Professor Lineu Prestes, 2242, São Paulo, SP 05508-000 Brazil; Center for the Study of Venoms and Venomous Animals (CEVAP), São Paulo State University (UNESP – Univ Estadual Paulista), Botucatu, São Paulo Brazil

**Keywords:** Gamma radiation, Structural modifications, Crotamine, Snake venom, *Crotalus durissus terrificus*

## Abstract

**Background:**

Since ionizing radiation has the potential to alter the molecular structure and affect the biological properties of biomolecules, it has been successfully employed to attenuate animal toxins. The present study aimed to characterize the structural modifications on irradiated crotamine, a toxin from *Crotalus durissus terrificus* venom, using circular dichroism (CD), fluorescence, Fourier transformed infrared spectroscopy (FTIR), atomic force microscopy (AFM) and differential scanning calorimetry (DSC).

**Methods:**

A combination of size exclusion and ion-exchange chromatography was used to purify the peptide using crude venom. The pure toxin was then submitted to 2 kGy gamma irradiation doses from a cobalt-60 source. Native and irradiated crotamine were analyzed using a fluorescence spectrophotometer. Wavelength was fixed at 295 nm and fluorescence emission scans were collected from 300 to 400 nm. CD and FTIR techniques were used to identify the secondary structure of both samples. DSC analyses were performed at a starting temperature of 20 °C up to a final temperature of 90 °C. AFM provided a 3D profile of the surfaces of both crotamine forms adsorbed on mica.

**Results:**

Fluorescence spectroscopy showed that the quantum yield of the irradiated form decreased. CD spectra of native and irradiated crotamine solutions showed differences between the samples in wavelength, indicating that irradiation induced a transition of a small portion of the random coil regions towards an α-helical conformation. FTIR and CD showed that the native and irradiated crotamine spectra were different with regard to secondary structure. The thermodynamic analysis showed that irradiation caused changes in the calorimetric profile and CD showed that temperature-induced changes also occur in the secondary structure. Finally, AFM showed the possible formation of insoluble aggregates.

**Conclusions:**

Our results indicate that irradiation leads to progressive changes in the structure of the toxin, which could explain a decrease in myotoxic activity.

## Background

Serum therapy is the only specific and effective treatment against snakebites. In Brazil, it consists of the administration of antivenom produced in immunized horses. However, the high toxicity of snake venom reduces their lifespan and thus hinders the production of antivenom [1–5].

Several methods of snake venom detoxification involve chemical or physical modification of toxins [6–13]. Gamma irradiation is a potential tool for venom detoxification, since the structural changes caused by this radiation slow down or stop enzymatic activities of toxic proteins, while retain epitopes that induce efficient immune responses [14–20]. Gamma rays are forms of ionizing radiation that interact with biomolecules in solutions through two processes. The direct process, whereby radiation reaches the target biomolecules, and the indirect process, in which water radiolysis products interact with the target molecules. The indirect process is the main way of interaction and accounts for approximately 80 % of the total effects [21, 22]. Previous studies showed that the most appropriate radiation dose for reducing venom toxicity and maintaining immunogenicity in order to increase the production of antiserum is 2 kGy [23–27].

Envenomation resulting from *Crotalus durissus terrificus* bites accounts for 14 % of all snake accidents in Brazil and has a high mortality rate [19, 28]. The structural effects of gamma rays on venoms and toxins are not yet fully understood and this study therefore aimed to characterize the effects of radiation on crotamine from *Crotalus durissus terrificus* venom.

Crotamine is a small basic polypeptide myotoxin (pI – 10.3) with a number of isoforms and a molecular weight of 4882 Da. It is composed of 42 amino acid residues without free sulfhydryl groups and reticulated by three disulfide bonds. This toxin induces skeletal muscle spasms in mammals leading to spastic paralysis of peripheral origin [29, 30].

In 2013, Coronado *et al*. [31] isolated a single isoform and determined the 1.7-Å-resolution crystal structure of crotamine, revealing distinct cationic and hydrophobic surface areas located on opposite sides of the molecule. The topological analysis classified the crystal structure as α1β1α2β2. Residues Lys2-Lys7 form a single α-helical turn which flanks a two-stranded antiparallel β-sheet formed from residues Gly9-Pro13 (β1) and Trp34-Lys38 (β2) located in the core of the molecule. A short α-helical turn is formed from residues Pro20-Ser23 [31].

Determining protein structure is crucial to understand their mode of action, thus contributing to the development of their potential as molecular tools or new drugs. Although a number of studies have already shown the biological properties of irradiated proteins, doubts remain on what refers to the structural modifications suffered due to irradiation and the resulting effects on the mode of action of crotamine [25, 26, 32, 33]. Since the crystal structure of crotamine has already been described, this study aims to investigate the radiation-induced conformational changes suffered by the peptide.

## Methods

### Venom

Crude lyophilized venom extracted from *Crotalus durissus terrificus* was supplied by the Center for the Study of Venoms and Venomous Animals (CEVAP) of São Paulo State University (UNESP) in Botucatu, SP, Brazil.

### Protein purification

Crude venom (70 mg) was dissolved in a 200 mM pH 3 ammonium formate buffer, centrifuged at 14,000 *g* for five minutes to remove insoluble material, and then fractionated using a Superdex 75 10/300 column (GE Healthcare, USA) equilibrated in the same buffer at a flow rate of 0.8 mL/minute. The absorbance of the eluate was monitored at 280 nm.

The crotamine fraction was pooled and refractionated using a 1 mL Resource S column (GE Healthcare, USA) equilibrated in 50 mM sodium phosphate buffer, pH 7.8 (buffer A). Buffer B was identical to buffer A, except that it was supplemented with 2.0 M NaCl. After an initial wash (5.0 mL) with 5 % buffer B, the protein was eluted with a linear gradient from 5 to 30 % buffer B. The fractions were pooled and then desalted by dialysis against water and lyophilized.

### Irradiation

The purified toxin was dissolved in 0.15 M NaCl to a final concentration of 2 mg/mL and submitted to 2 kGy gamma irradiation doses from a cobalt-60 (^60^Co) source (Gammacell 220, Canada) in the presence of atmospheric oxygen, at room temperature and with a dose rate of 1.2 kGy/h.

### Fluorescence Quenching

After measuring absorbance at 280 nm of both samples to ensure that irradiation did not cause any alteration in peptide concentration, 100 μg/mL aliquots in PBS buffer (pH 7.2) of the native and irradiated crotamine were analyzed at 25 °C using a Varian Cary Eclipse fluorescence spectrophotometer (Varian Australia Pty Ltd, Australia). The excitation wavelength was fixed at 295 nm and fluorescence emission scans were collected from 300 to 400 nm.

### Circular Dichroism Spectroscopy (CD)

Circular dichroism spectroscopy was used to quantify the secondary structure of the native and irradiated toxin using 500 μL of each sample at 100 μg/mL, placed in quartz cuvettes with a 1 mm path length using a Jasco J-810 L CD Spectropolarimeter (Japan). Spectra were measured between 190 nm and 260 nm.

Three runs were performed for each sample and the data were processed subtracting the blank spectrum. The temperature was increased in increments of 10 °C from 20 °C to 90 °C throughout the experiment. The alteration of structural elements was quantified using the K2d software (http://kal-el.ugr.es/k2d/spectra.html).

### Fourier Transform Infrared Spectroscopy (FTIR)

The vibrational spectra of the molecules were analyzed using FTIR to determine the secondary structure of native and irradiated crotamine. The samples were scanned over the range 650 to 2,000 cm^−1^ at a resolution of 4 cm^−1^ and 120 scans. The spectra were baseline corrected and normalized to the amide band I to minimize homogeneity of variance. The area of each band was calculated based on the method used by Naumann [34, 35].

### Differential Scanning Calorimetry (DSC)

DSC was performed using a Mettler Toledo Differential Scanning Calorimeter 822e after diluting the native and irradiated crotamine samples to 400 μg/mL in a 25 mM pH 7.2 phosphate buffer. The thermodynamic analysis was carried out by gradually heating the samples (1 °C/min) from a starting temperature of 20 °C to a final temperature of 90 °C. The signal was registered every 30 s and corrected against the buffer spectrum.

### Atomic Force Microscopy (AFM)

AFM provides a 3D profile of the surface on a nanoscale, by measuring forces between a sharp probe (<10 nm) and a surface at a very short distance (0.2-10 nm probe-sample separation). The native and irradiated crotamine were diluted to 0.01, 0.1 and 1 μg/mL in phosphate buffer (10 mM KH_2_PO_4_, 150 mM KCl, pH 6). A drop of approximately 40 μL of crotamine solution was deposited on a pre-prepared mica support which was incubated for between 15 and 20 min. Mica surfaces are commonly used for obtaining topographic images of proteins using AFM because they are flat, hydrophilic, and because mica has a high affinity to protein molecules [36]. Each sample was then washed three times in deionized water. This entire process was performed in a laminar flow to avoid contamination of the sample surface. The experiment was conducted at room temperature in tapping mode, in which the cantilever oscillates at its resonance frequency or very close to contact.

## Results and discussion

Crotamine was isolated using a two-step chromatography procedure: size exclusion followed by ion exchange. The identity and homogeneity of the peptides were confirmed using mass spectrometry which detected three crotamine isoforms due to the low resolution of the ion exchange column used in the second purification step. The molecular mass of the most abundant isoform was 4883.2548 Da (data not shown).

**Fig. 1:**
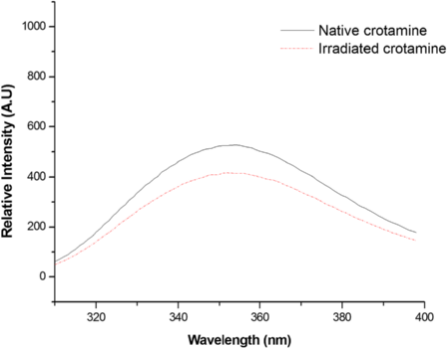
Fluorescence spectra of native and irradiated crotamine. The native form of crotamine is represented by a solid line and the irradiated form by the dashed line. The results show that irradiated crotamine quantum yield decreased by about 25 %

Irradiation of aqueous solutions induces chemical and structural alterations in proteins and peptides related to an attenuation or abolishment of biological activity and interferences in immunological properties [14–20]. It is known that tryptophan fluorescence is strongly influenced by its local environment. Thus, changes in protein conformation influence spectrum data. The quenching spectra of the solvent-mediated fluorescence of native and irradiated crotamine can be observed in Fig. 1. The analysis of fluorescence quenching showed that irradiated crotamine quantum yield decreased about 25 % in comparison to the native form. This suggests an increase in the solvent accessibility of tryptophan, possibly due to unfolding of the polypeptide chain, since exposure to ionizing radiation is known to alter molecular structure. Coronado *et al.* [31] have suggested that solvent exposure of hydrophobic residues makes crotamine unusually ‘sticky’. Most hydrophobic residues are located on one side of the molecule and the positively charged residues are clustered on the opposite side. These residues are exposed on the surface of crotamine and form distinct hydrophobic and cationic areas that are positioned roughly on opposite sides of the amphiphilic molecule [31]. The decrease of fluorescence in irradiated toxins can be explained by an increase in the solvent accessibility of the tryptophan, possibly due to unfolding of the polypeptide since exposure to ionizing radiation changes molecular structure. In the case of crotamine, the decrease was not expressive because most of the hydrophobic residues are already exposed in the native form of the toxin.

Although fluorescence can be a very helpful indicator of molecular change, when it comes to changes in secondary structure, such measurements are difficult to interpret directly (α-helix and β-sheet). When the structure of native protein containing α-helical and β-sheet areas is denatured it undergoes an unfolding process. Often, the fraction of non-covalent bonds in α-helical, β-sheet and aperiodic conformations may be estimated using highly sensitive measurements, such as circular dichroism (CD) and infrared (IR) spectroscopy. In favorable cases, the spectral effects of conformational changes caused by physical and biological phenomena may be observed [37].

**Fig. 2:**
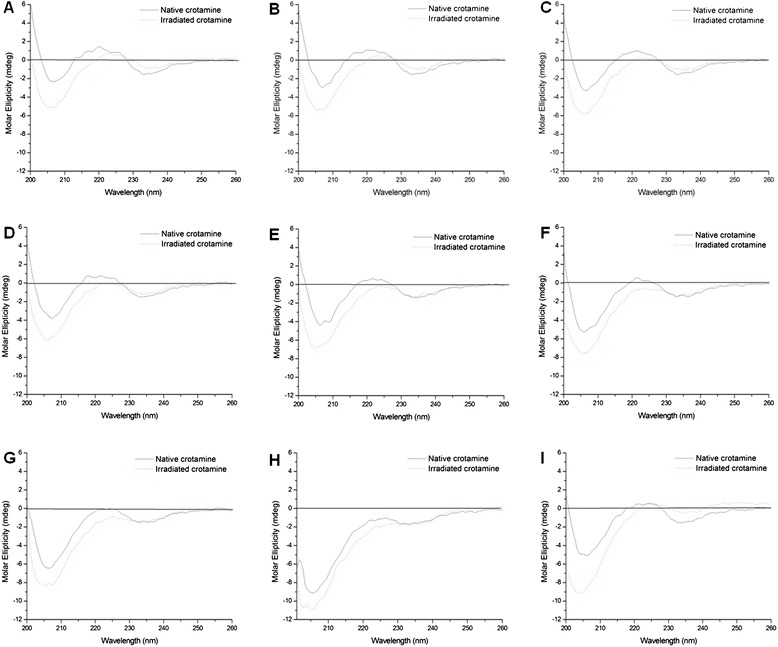
CD spectra of native and irradiated crotamine. (**a**) At room temperature. (**b**) CD spectra at 30 °C. (**c**) CD spectra at 40 °C. (**d**) CD spectra at 50 °C. (**e**) CD spectra at 60 °C. (**f**) CD spectra at 70 °C. (**g**) CD spectra at 80 °C. (**h**) CD spectra at 90 °C. (**i**) CD spectra at 20 °C after heating. Structural changes due to thermal denaturation changed the CD spectra

Figure 2a shows the CD spectra of native and irradiated crotamine at room temperature. With regard to the native toxin (solid line), the relative proportions of β-sheet and α-helix were 4 % and 48 %, respectively, while, for the irradiated peptide (dashed line), the α-helix content was estimated at 8 %, with a corresponding decrease of 4 % of random coil. In the α-helical protein, a negative band near 222 nm is observed due to the strong hydrogen-bonding environment of this conformation. A second transition at 190 nm is split into a negative band near 208 nm and a positive band near 192 nm. The CD spectra of native crotamine shows a negative band near 208 nm, indicating the presence of α-helical conformation.

Pelton and McLean [37] elucidated that short helices can result in reduced bands in the spectra. Coronado *et al.* [31] illustrated that both α-helix structures in crotamine are formed by few residues, with a single turn in α2 and two turns in α1. The CD spectra of β-sheets display a negative band near 216 nm, a positive band between 195 and 200 nm, and a negative band near 175 nm. However, the position and magnitude of these bands is variable, resulting in less accurate predictions for β-structure than for α-helices using CD [37]. Our data indicate that radiation induced the transition of a small portion of the random coil regions towards an α-helical conformation. By using a secondary structure prediction tool, we observed that a region containing three cysteines (D24-K38) is prone to forming α-helices [38]. It is possible that this does not occur in the native peptide as these residues form disulfide bonds that stabilize the molecule. Irradiation may cause disruption in these reticulation bonds, enabling the formation of a non-native helical domain.

We also performed CD spectroscopy with temperature changes in an attempt to assess modifications in the thermal stability of crotamine after exposure to radiation. Initially, the temperature was maintained at 20 °C (Fig. 2a) and subsequently increased by 10 °C every 30 min until a final temperature of 90 °C was reached (Fig. 2b to h). We observed that structural changes due to thermal denaturation lead to changes in CD spectra and it seems that complete denaturation occurred at 90 °C. After reaching the maximum temperature, we reduced the sample temperature to 20 °C (Fig. 2i) and took a new reading which showed only a partial recovery of structural elements. Irradiated crotamine displayed partial denaturation and it is apparent that this form of the toxin was more sensitive to temperature since at 70 °C only random conformation could be observed.

Differential scanning calorimetry (DSC) is extremely useful for characterizing temperature- induced conformational changes in proteins, especially with respect to the factors associated with protein stability [39]. The thermodynamic analysis showed that irradiation modified molecule enthalpy. Endothermic transitions decreased while exothermic transitions increased.

**Fig. 3:**
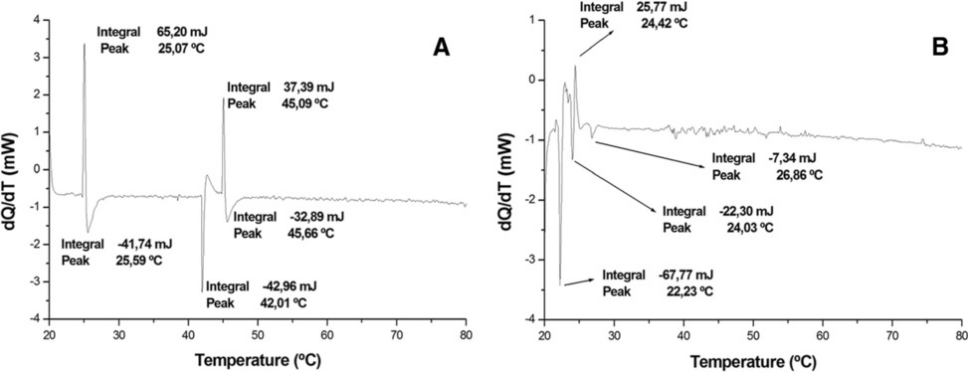
The thermodynamic analysis of (**a**) native and (**b**) irradiated crotamine using DSC showed an upward peak (structure formation) and downward peak (structure melting) in native crotamine at approximately 25 °C and 45 °C, respectively. Irradiated crotamine showed an irregular spectrum with a first component which indicates that the irradiated toxin protein melted at 22.23 °C, suggesting lower enthalpy

Figure 3a shows native crotamine with an upward peak at approximately 25 °C, followed by a downward peak at 45 °C. These data suggest that structure formation and subsequent melting occurred. Furthermore, the results indicate another, probably intermediate, state of transition at around 42 °C. It may also be inferred that the toxin becomes completely denatured at 46 °C.

Figure 3b shows an irregular spectrum with a first component indicating that the irradiated toxin protein melted at 22.23 °C, which reflects lower enthalpy in the irradiated sample. In other words, the energy required to denature the toxin exposed to radiation is much lower, showing that structural stability was significantly affected by the irradiation.

Infrared spectroscopy is a well-established experimental procedure used to analyze the secondary structure of polypeptides and proteins associated with the vibration of repeating structural units, especially the frequency at which amine bonds absorb infrared radiation. The use of the infrared spectroscopy technique to assess protein secondary structure has undergone a renaissance with the development of Fourier transform infrared spectroscopy.

Nine normal modes are allowed for the amide band of proteins in order of decreasing frequency: A, B, and I-VII. The amide band I (80 % C = O stretch, near 1650 cm^−1^) is the most commonly used to assign secondary structures to proteins [37].

**Fig. 4:**
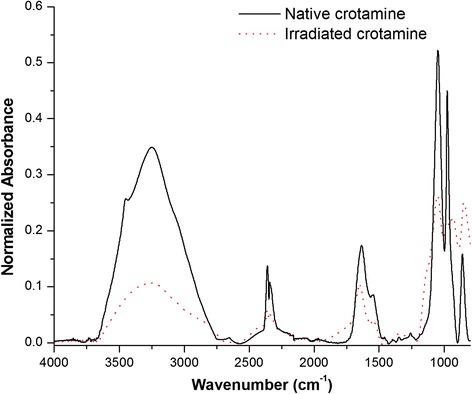
FTIR spectra of the native (solid line) and irradiated (dotted line) forms of crotamine. The curve of the native crotamine differs substantially from that of the irradiated sample, exemplified by a significant discrepancy in the spectral region of the amide I band

Figure 4 shows the FTIR spectra of crotamine, both in its native (solid line) and in irradiated (dotted line) forms. These spectra show all amide bands of crotamine: amide band I (near 1650 cm^−1^), II (60 % N-H bend and 40 % C-N stretch, near 1550 cm^−1^), and III (40 % C-N stretch, 30 % N-H bend, near 1300 cm^−1^). We observed that the native crotamine curve differs substantially from that of the irradiated sample, with particularly significant differences in the spectral region of the amide band I, indicating drastic changes in secondary structure. We also observed that the region between 3500 and 3200 cm^−1^ was different in the two forms of crotamine. Although this range is not particularly significant when it comes to the study of the secondary structure, it is important to note that the ~ 3500 cm^−1^ region corresponds to the OH bonds of hydroxyl groups and the ~ 3200 cm^−1^ region corresponds to the NH of the amide grouping links [40]. All proteins are made up of covalently linked amino acid sequences. These compounds have -OH and -NH groups which have the ability to form a strong network of intermolecular bonds which gives rise to the tertiary structure of the protein, i.e., its spatial conformation. Thus, the FTIR results suggest that the tertiary structure of crotamine has changed since absorbance was lower in the curve of the irradiated sample.

**Fig. 5:**
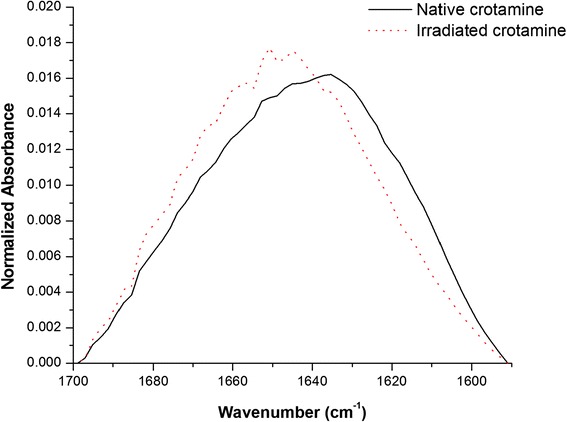
Spectra of native and irradiated crotamine in the spectral region of the amide band I. The spectra of native crotamine are represented by a smooth curve with peaks along the amide I region, while the irradiated crotamine curve shows well defined peaks

Due to the particular importance of the characteristic bands of the amide region I aforementioned, this area is zoomed in Fig. 5. With respect to the irradiated protein, it can be observed that there is a shift in band peaks and an increase in the intensity of some peaks (for example regions 1, 2 and 3) which could be ascribed to changes in the secondary structure of crotamine. Proteins known to adopt an α-helical conformation have strong amide I bands between 1650 and 1655 cm^−1^. The hydrogen-bonding strength of β-sheets is more variable due to their flexibility and tendency to twist. Although a strong band between 1612 and 1640 cm^−1^ and a weaker band at about 1685 cm^−1^ are common in β-sheets, weak bands at somewhat lower frequencies (1665–1670 cm^−1^) have also been observed [37].

In Fig. 5 the spectra of native crotamine are presented as a smooth curve with peaks along the amide I region, while well-defined peaks appear on the curve of the irradiated crotamine. It is evident that the regions most affected by radiation were the spectral regions corresponding to the α-helix (1650 and 1655 cm^−1^), since major differences were observed at these wave numbers. It was also observed that the curve of the irradiated crotamine shifted approximately 20 cm^−1^ to the left, suggesting lower stability in this protein, corroborating the results of the DSC. Given the effects of radiation associated with the ionization of the molecule and its ability to modify the protein structure, these phenomena may be associated with a strong interaction between protein products of lower molecular mass and may demonstrate the formation of peptides, amino acids or even protein aggregates [17, 41]. The presence of these molecular neighbors may modify the interaction of the peptide groups, changing the total energy of the molecule.

The quality of data from atomic force microscopy (AFM) used for imaging and manipulating biomolecules has improved due to advances in equipment, sample preparation and image acquisition conditions. This is the only instrument that can image samples in aqueous solution at the subnanometer scale [42].

**Fig. 6:**
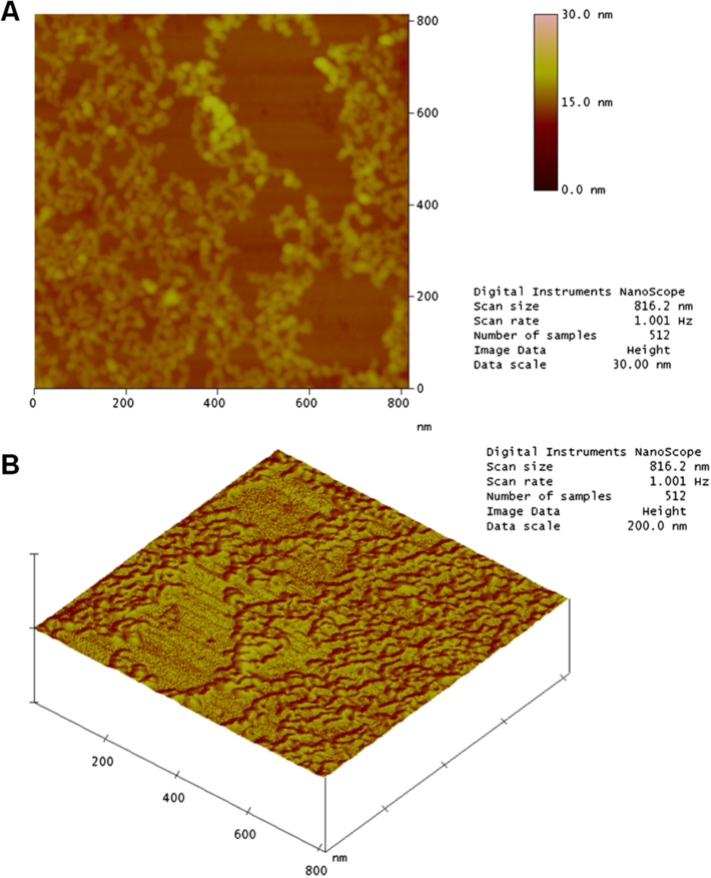
AFM image of native crotamine showing the (**a**) structure profile and (**b**) topographical profile. The mica surface is completely flat in the image. The other sides are covered with adsorbed crotamine

Figure 6a shows a topographic image of a mica surface with a sample of native crotamine at a concentration of 0.1 g/mL. It is possible to observe small well-differentiated structures and well-defined rounded “chains”. This is because the mica surface is extremely flat. There are also regions where the toxin was not adsorbed. Figure 6b illustrates the results for native crotamine.

**Fig. 7:**
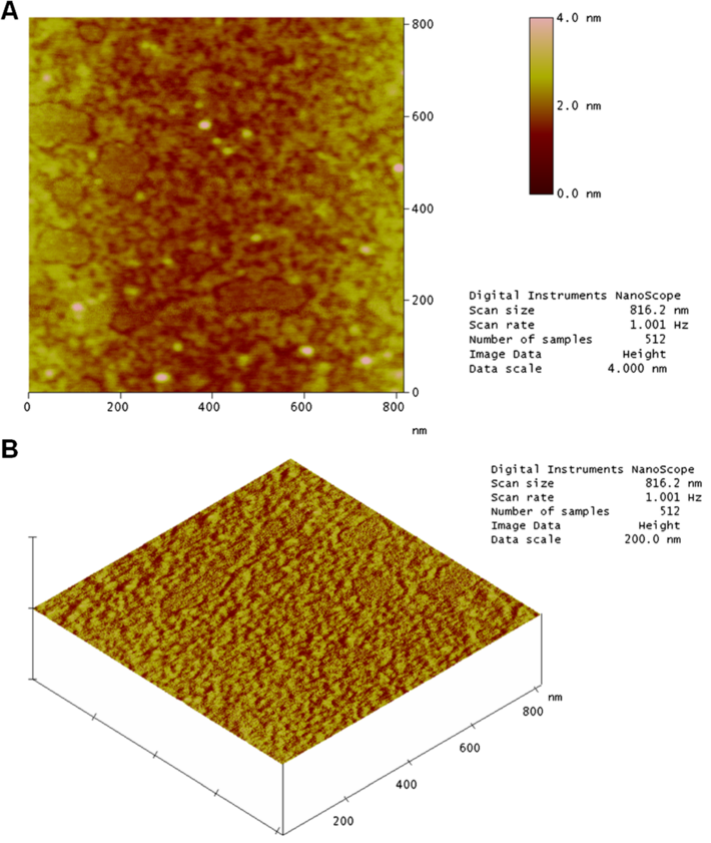
AFM image of irradiated crotamine showing the (**a**) structure profile and (**b**) topographical profile. The mica surface was completely covered by irradiated crotamine

The AFM image in Fig. 7a shows that the profile for irradiated crotamine at the same concentration is different. It is not possible to observe the flat surface of the mica, showing that the irradiated sample covered the entire area, suggesting alterations in the distribution of charged residues on the surface of the molecule. Furthermore, the chain structures identified in the native sample were not observed. These results suggest a change in the conformational structure of crotamine. Exposure to radiation can break the carbon backbone and create smaller fragments that may be reorganized forming aggregates which are invisible to the naked eye. The topographical profile for irradiated toxin was obtained in the same way (Fig. 7b).

## Conclusions

Based on our findings, we conclude that crotamine suffered conformational changes after being exposed to 2 kGy gamma radiation doses from a cobalt-60 source.

The fluorescence analysis allowed us to observe small changes in the tertiary structure of the protein, shown by quenching of the tryptophan fluorophores which are partially exposed in the native molecule.

The results of circular dichroism and infrared spectroscopy showed that there were changes to the secondary structure of the toxin. Such changes in the secondary structure of proteins may be related to an attenuation or abolishment in its biological activities, such as decreased myotoxic activity. This fact has been demonstrated by previous studies [14–17].

Subjecting the samples to changes in temperature was also used as a mean of comparing the thermal stability of the native and irradiated toxin. Circular dichroism and differential scanning calorimetry showed that irradiation induces energy (enthalpy) changes in the peptide, making it more susceptible to thermal denaturation. AFM reinforces these observations, showed by the formation of “soluble aggregates” in the irradiated sample.

Taken as a whole, our data indicate that the irradiation of crotamine in solution leads to significant and apparently homogeneous conformational changes, with alterations of structural elements and a loss of enthalpy, resulting in reduced structural stability. The use of crotamine to investigate the impact of gamma ray exposure on peptides provides important insights into the potential of radiation as a tool for attenuating toxins and contributing to the design of safer antigens for vaccine and antiserum production.

## Abbreviations

CD: Circular dichroism
FTIR: Fourier transformed infrared
AFM: Atomic force microscopy
DSC: Differential scanning calorimetry
